# Innovative approach to monitor performance of integrated disease surveillance and response after the Ebola outbreak in Sierra Leone: lessons from the field

**DOI:** 10.1186/s12913-022-08627-6

**Published:** 2022-10-20

**Authors:** Charles Njuguna, Mohamed Vandi, James Sylvester Squire, Joseph Sam Kanu, Wilson Gachari, Evans Liyosi, Jane Githuku, Alexander Chimbaru, Ian Njeru, Victor Caulker, Malimbo Mugagga, Stephen Sesay, Ali Ahmed Yahaya, Ambrose Talisuna, Zabulon Yoti, Ibrahima Socé Fall

**Affiliations:** 1 World Health Organization Country office, 21 A & B Riverside Drive, off King Harman Road Brookfield, Freetown, Sierra Leone; 2 Ministry of Health and Sanitation, Freetown, Sierra Leone; 3 World Health Organization Regional Office for Africa, Brazzaville, Congo; 4 World Health Organization, Geneva, Switzerland

**Keywords:** Public Health, Integrated Disease Surveillance and Response, Monitoring, Technology

## Abstract

**Background:**

Supervision of healthcare workers improves performance if done in a supportive and objective manner. Regular supervision is a support function of Integrated Disease Surveillance and Response (IDSR) strategy and allows systematic monitoring of IDSR implementation. Starting 2015, WHO and other development partners supported the Ministry of Health and Sanitation (MoHS) to revitalize IDSR in Sierra Leone and to monitor progress through supportive supervision assessments. We report on the findings of these assessments.

**Methods:**

This was a cross-sectional study where six longitudinal assessments were conducted in randomly selected health facilities. Health facilities assessed were 71 in February 2016, 99 in July 2016, 101 in May 2017, 126 in August 2018, 139 in February 2019 and 156 in August 2021. An electronic checklist based on selected core functions of IDSR was developed and uploaded onto tablets using the Open Data Kit (ODK) platform. Supervision teams interviewed health care workers, reviewed documents and made observations in health facilities. Supervision books were used to record feedback and corrective actions. Data from the supervisory visits was downloaded from ODK platform, cleaned and analysed. Categorical data was summarized using frequencies and proportions while means and medians were used for continuous variables. Z test was used to test for differences in proportions.

**Results:**

Completeness of IDSR reporting improved from 84.5% in 2016 to 96% in 2021 (11.5% points; 95% CI 3.6, 21.9; *P*-value 0.003). Timeliness of IDSR reports improved from 80.3 to 92% (11.7% points; 95% CI 2.4, 22.9; *P*-value 0.01). There was significant improvement in health worker knowledge of IDSR concepts and tools, in availability of IDSR standard case definition posters and reporting tools and in data analysis practices. Availability of vaccines and temperature monitoring tools in health facilities also improved significantly but some indicators dropped such as availability of IDSR technical guidelines and malaria testing kits and drugs.

**Conclusion:**

Supervision using electronic tool contributed to health systems strengthening through longitudinal tracking of core IDSR indicators and other program indicators such as essential malaria commodities and availability and status of routine vaccines. Supervision using electronic tools should be extended to other programs.

## Background

Supportive supervision is a unique approach to monitoring performance through face to face interactions between a skilled health care worker and a less skilled worker [1]. First promoted in the era of primary health care, supportive supervision sought to improve performance of healthcare workers offering essential services such as immunization in remote areas [2]. However, this approach can be impeded by poor coordination, lack of motivation, geographical barriers, competing activities, high costs and armed conflicts [1, 3].

Initially, a top-down approach to supervision was used with inspection and correction of junior workers done by a senior officer. There has been a shift to a more collaborative approach (support supervision) with focus on identifying challenges in service delivery and involving the provider in finding solutions. This approach improves motivation and ownership by the health care providers [4]. Records reviews, direct observations, use of objective indicators, collaborative problem solving and targeted on job training are additional collaborative approaches used [1, 5].

In 1998, the World Health Organization (WHO), Africa Regional Office adopted the Integrated Disease Surveillance (IDS) strategy, later renamed Integrated Disease Surveillance and Response (IDSR) strategy to strengthen public health surveillance and response in member states [6]. Implementation of the IDSR strategy varies, with the initial momentum exhibited in the first years of implementation declining over time due to several challenges such as non-sustainable financial resources, poor coordination, inadequate training, high turnover of staff, inadequate supervision from the next level, erratic feedback, weak laboratory capacities, unavailability of job aids and poor availability of communication and transport systems particularly at the periphery [7–9]. Considering the growing threat of epidemics especially from new and emerging pathogens, the need for strengthened surveillance systems has never been greater. Ministries of Health in WHO member states are urged to monitor implementation of IDSR as this directly contributes to fulfillment of the International Health Regulations (2005) [10]. National level health staff utilize supervision visits to monitor the implementation of Integrated Disease Surveillance and Response (IDSR) activities and identify key areas that need improvement [11]. While training of sub- national health care workers on IDSR provides them with basic understanding of public health surveillance, supportive supervision reinforces concepts, and identifies and resolves challenges affecting implementation [12].

### The effect on Ebola Virus Disease outbreak on the health workforce in Sierra Leone

Health care workers are a high-risk group for highly infectious diseases such as Ebola Virus Disease (EVD). Out of 304 health care workers infected with Ebola virus in Sierra Leone in 2014–2016, two hundred and twenty-one (221) died and several others resigned out of fear of getting infected. This reduced the ratio of skilled health workers from 17.2/ 10,000 to < 4 /10,000 population [13–15]. After the outbreak, the Ministry of Health and Sanitation (MoHS) embarked on strengthening health systems, improving public health surveillance and cross border surveillance [14].

### Improving public health surveillance after the Ebola outbreak

Towards the end of the Ebola Virus Disease outbreak, the Sierra Leone Ministry of Health and Sanitation in collaboration with World Health Organization country office (WCO) and other health sector development partners, embarked on revitalization of IDSR. This involved adaptation of the 2010 WHO-Africa region IDSR technical guidelines and training modules, training of healthcare workers and providing inputs and infrastructure to support IDSR implementation [16].

Previously, paper-based checklists were used during supportive supervision in Sierra Leone and health facilities were selected purposively based on ease of access. Thus, it was likely that health facilities in remote areas were omitted from supervisory visits. Collation and analysis of data from the supervisory visits was seldom done as it required abstraction of data from paper-based checklists to a computer and this often required hiring of data clerks due to staff shortage. To overcome these issues, we adopted an electronic supervision checklist and an open source platform to collect and manage data. This paper describes the innovative, integrated approach to IDSR supportive supervision. We share the lessons learnt, hopeful that they can provide insight for improving monitoring of public health surveillance programs particularly for countries implementing IDSR.

The integrated support supervision process described in this paper is considered an innovative way of monitoring IDSR performance indicators for several reasons. First, routine supervision visits were used to collect comprehensive and representative data to monitor key IDSR indicators from 2016 to 2021. During the visits, verbal and written feedback on performance was then given to the health facilities and district health management teams which ensured that corrective action was taken where necessary. Second, an integrated questionnaire was used which not only collected surveillance data, but also data from other related programs such as laboratory, immunization and malaria. Third, the use of electronic supervision checklist and an open source data collection platform to collect and manage the data ensured secure storage and rapid retrieval of data for analysis which enabled monitoring of performance over time.

## Methods

### Study **setting and design**

This cross-sectional study was part of operational research to monitor implementation of IDSR program in Sierra Leone post Ebola outbreak. Data was collected through supportive supervision visits to health facilities across all districts in Sierra Leone from 2016 to 2021. The data collected was used to monitor and improve IDSR implementation in the country.

### Selection of health facilities

The total number of health facilities sampled nationally (sample size) per each support supervision visit was purposively selected based on available resources. There were about 1425 health facilities in the country in 2021, distributed by type as follows, in order of size and scope of services offered (from largest to smallest): Hospital 4%, Community Health Centres (CHC) 19%, Community Health Posts (CHP) 30%, Maternal and Child Health Posts (MCHP) 43% and Clinic 3%. About 10% of the total existing facilities per year was sampled in each visit. To ensure representativeness, health facilities were selected from each type of health facility (except clinics which are mostly private) in all the districts. Selection of health facilities in each district was done randomly from a list of health facilities stratified by health facility type as indicated above. First, health facilities were stratified according to the type of health facility. Thereafter, random sampling, using computer generated numbers was done to identify at least one hospital, three CHC, three CHP and three MCHP in each district. Where available, major private health facilities equivalent to a hospital were included.

**Fig. 1:**
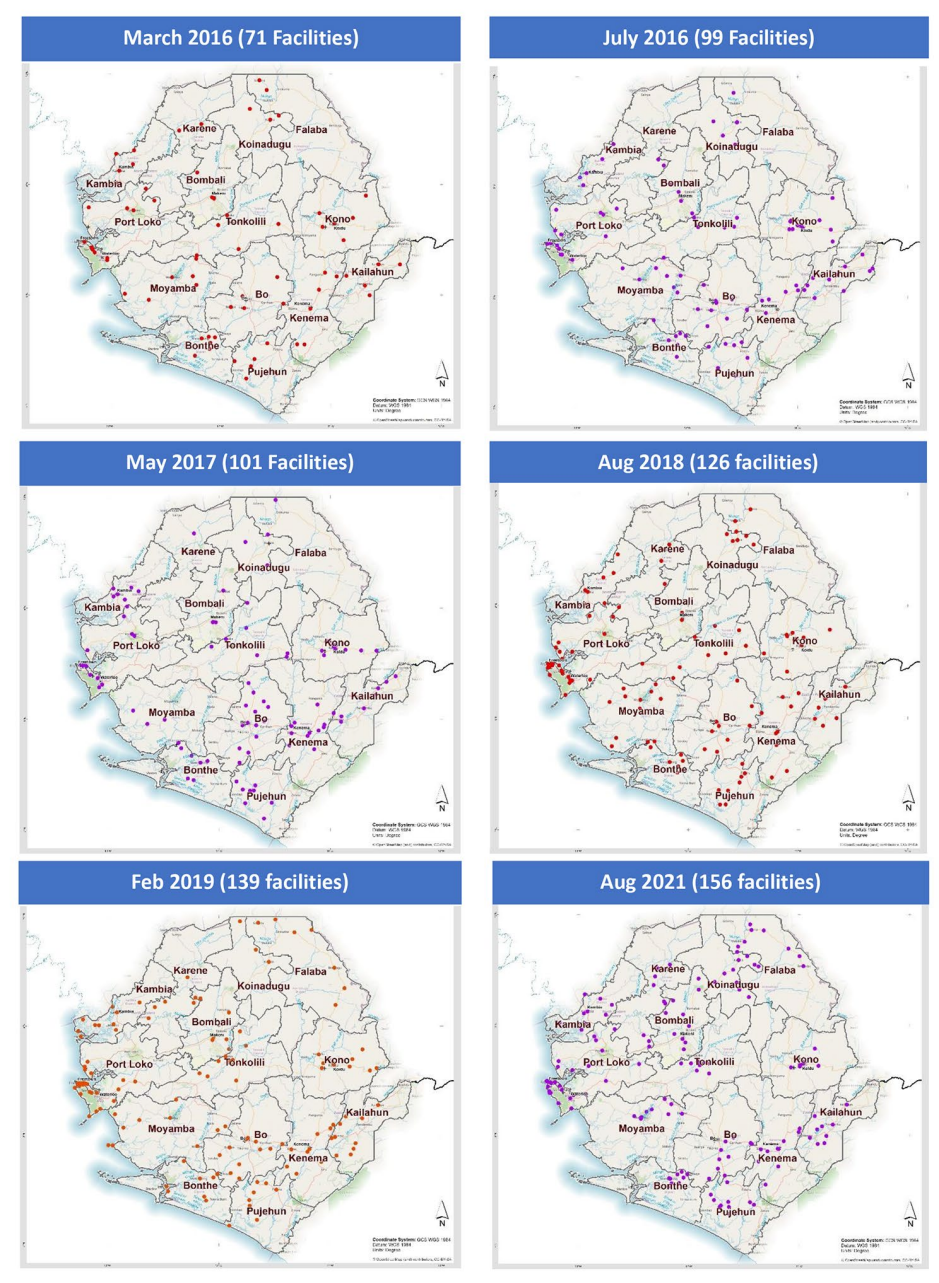
Map of Sierra Leone showing health facilities visited during support supervision, 2016–2021

Over the six-year period, six national level supportive supervision visits were conducted covering 71 health facilities in February 2016, 99 in July 2016, 101 in May 2017, 126 in August 2018, 139 in February 2019 and 156 in August 2021 (Fig. 1). No national level supervisory visit was carried out in 2020 due to COVID-19 pandemic. Apart from the national supervision visits, the District Health Management Teams conducted two to four supervision visits per year for the facilities under their jurisdiction. However, this data was captured in a separate database and is therefore not included in this article.

### Data collection

The assessment teams comprised of national level staff from the surveillance and laboratory programs of MOHS as well as development partners like World Health Organization who were supporting the supervision missions technically and financially. Respondents in the assessments were a team of healthcare workers involved in surveillance and administrative functions in the health facilities. These included the IDSR focal person, health facility in charge, laboratory technician and the nurse in charge of vaccination activities.

One week prior to each supportive supervisory visit, the MoHS through the National Disease Surveillance Program contacted the District Medical Officers (DMO) and District Surveillance Officers (DSOs) to inform them of the upcoming supervisory visits. On the first day of the visit, the supervisory team comprising of officials from MoHS and supporting development partners met with the District Health Management Team (DHMT) members to discuss the purpose of the supervisory visits and to select health facilities for the assessment. The selected health facilities were not informed of the visit in advance in order to avoid pre-emptive interference with data tools. Upon reaching a health facility, the supervisory team captured the geographic coordinates of the health facilities using automatically generated geo- coordinates. Recording of geo-coordinates for each facility ensured that there was evidence of representativeness in the distribution of selected health facilities. Next the team conducted interviews, verified documents and confirmed availability of IDSR reporting tools.

Data was collected in real-time using a structured electronic questionnaire created using Open Data Kit platform and loaded onto tablets. Using the electronic tool eased data collection and management, making it possible to analyse data, provide timely feedback to the health facilities staff, and also track performance over time. The questionnaire covered six out of eight core functions of IDSR as well as an assessment on related programmatic areas such as available laboratory services, maternal deaths, malaria commodities and routine immunization services. The questionnaire collected information on (1) case identification; (2) reporting of priority diseases; (3) data analysis and interpretation; (4) investigation and confirmation of reported cases and outbreaks; (5) feedback between the district management teams and health facilities; and (6) monitoring and evaluation (Table 1).

**Table 1:** IDSR Core Functions Assessed During Integrated Support Supervision, Sierra Leone, 2016–2021

	Core Function	Assessment methods	Main focus areas
1	Identification of priority diseases, conditions and events	Interview, observation, review of registers	Assessed the ability of healthcare workers to use standard case definition to identify priority diseases through records review and active case search in the community
2	Reporting of Priority diseases	Interview, documents review, observation	Checked if HCWs are aware of reporting requirements for priority diseases and the availability and use of reporting tools
3	Data analysis and interpretation	Interview and observation	Checked if data analysis and interpretation was done at the facility
4	Investigation and confirmation of outbreaks	Interview	Assessed if HF are able to detect outbreaks from various sources, notify the district and participate in outbreak investigations
5	Feedback	Interview	Confirmed if the HF gets feedback on IDSR performance from the DHMT
6	Monitoring and Evaluation	Interview, document review	Assessed the frequency of supervision done by the DHMT and mechanisms for monitoring IDSR performance in the HF

IDSR: Integrated Disease Surveillance and Response; HCW: Healthcare workers; HF: Health Facility; DHMT: District Health Management Team

The teams began by assessing availability of IDSR focal persons in the health facilities and their participation in district monthly review meetings. This is because the focal persons are important in coordinating IDSR activities. On case identification, supervisors checked if IDSR posters with case definitions were available and displayed in the consultation rooms. They then interviewed the respondents to gauge their knowledge of case definitions for five selected IDSR priority diseases/conditions (Acute flaccid paralysis, Neonatal tetanus, Measles, Cholera and Ebola Virus Disease). Additionally, COVID-19 was added in the 2021 assessment. A healthcare worker who mentioned all signs and symptoms correctly was classified as having adequate knowledge, while one who mentioned some of the signs and symptoms was classified as having limited knowledge. A healthcare worker who did not mention any of the appropriate signs and symptoms was classified as having no knowledge.

The section on reporting of priority diseases assessed knowledge of and compliance with reporting requirements for priority diseases. Copies of IDSR weekly reports for the previous four weeks were checked to confirm if all reports were available and if they had been submitted on time. The team also checked the availability of case-based reporting forms, weekly reporting forms, line listing forms, and rumour logbooks for recording suspected outbreaks/events. The section on data analysis assessed the capacity and type of data analysis conducted to monitor trends of priority diseases at the health facilities. The section on investigation captured information on case investigation and confirmation of reported outbreaks. The section on feedback assessed feedback mechanisms from the district to the health facilities and vice versa. It included a review of previous supervision reports to elicit frequency of supportive supervision by DHMT. The section on monitoring and evaluation assessed mechanisms for monitoring IDSR performance in the health facility.

Additional questions on immunization, malaria treatment commodities and maternal mortality were also included in the questionnaire. The questionnaire had compulsory fields and intrinsic data validation mechanisms to enhance data accuracy and completeness. Finally, at the end of the data collection process, the teams used supervision books available in each health facility to record strengths, gaps, challenges and recommendations agreed upon during the supervisory visit. These records were useful for follow up during subsequent supervisory visits. Supervision also provided an opportunity for on the job training and distribution of IDSR reference materials.

### Data analysis

Data was extracted from the Open Data Kit (ODK) platform first onto an MS Excel database, merged, checked for duplicates and completeness and then exported to Epi Info for analysis. We calculated means and medians for continuous variables and proportions for categorical data. Two proportion *Z-*test was used to compare differences in performance recorded during the baseline in February 2016 and the end line in August 2021. District weekly report completeness was defined as the proportion of health facilities that submitted required reports to the district health office. This was verified by checking if all copies of the IDSR weekly reports for the preceding four weeks were available. Timeliness was defined as the proportion of health facilities that submitted IDSR weekly reports to the district by 12 pm every Monday.

## Results

### Coordination of IDSR activities in health facilities

IDSR focal persons were present in most health facilities during all assessments. When compared to the baseline assessment, the proportion of IDSR focal persons who participated in the monthly district management team meetings increased significantly from 74.6% to 2016 (baseline) to 96.1% at end line in 2021 (increase of 21.5% points; 95% CI (11.8, 32.9); P-value < 0.0001). Conversely, availability of IDSR technical guidelines at the health facilities declined from 97.2% to 2016 to 82.1% in 2021 (decrease of 15.1% points; 95% CI (6.5, 22.2); P-value 0.002) (Table 2). The decline in availability of IDSR technical guidelines was mainly due to loss without replacement.

**Table 2:** Comparison of IDSR indicators in selected health facilities, Sierra Leone, 2016–2021

Core Function	Indicators	Feb_2016	July_2016	May_2017	Aug_2018	Feb_2019	Aug_2021	End Line vs. Baseline	P value
		N = 71 n (%)	N = 99 n (%)	N = 101 n (%)	N = 126 n (%)	N = 139 n (%)	N = 156 n (%)	Difference (95 CI)	
Coordination	Health facility (HF) has IDSR focal person	69 (97.2)	97 (98.0)	100 (99.0)	121 (96.0)	135 (97.1)	153 (98.1)	0.9 (-3.2, 7.9)	0.67
	HF IDSR focal person attends district monthly meetings	53 (74.6)	87 (87.9)	93 (92.1)	118 (93.7)	132 (95.0)	150 (96.1)	21.5 (11.8, 32.9)	**< 0.0001**
	HF has IDSR Technical guidelines	69 (97.2)	89 (89.9)	101 (100.0)	102 (81.0)	123 (88.5)	128 (82.1)	15.1 (6.5, 22.2)	**0.002**
Case Identification	Standard Case definition poster displayed in at least one location in the health facility	54 (76.1)	84 (84.8)	89 (88.1)	114 (90.5)	127 (91.4)	148 (94.9)	18.8 (9.2, 30.2)	**< 0.0001**
	IDSR focal person conducts active case search at least once a week	56 (78.9)	54 (54.5)	34 (33.7)	100 (79.4)	75 (54.0)	123 (78.8)	0.1 (-12.1, 10.7)	0.99
Reporting of Priority diseases	HCW correctly defines epidemiologic week	51 (71.8)	93 (93.9)	99 (98.0)	108 (85.7)	132 (95)	151 (96.8)	25 (15.0, 36.5)	**< 0.0001**
	HCW correctly defines zero reporting	67 (94.4)	94 (94.9)	99 (98.0)	121 (96.0)	138 (99.3)	147 (92.2)	2.2 (-6.4, 8.5))	0.55
	HCW knows the deadline for submitting weekly IDSR reports	65 (91.5)	96 (97.0)	100 (99.0)	115 (91.3)	138 (99.3)	151 (96.8)	5.3 (-0.8, 14.3)	0.09
	Weekly reporting forms available in HF	63 (88.7)	89 (89.9)	96 (95.0)	119 (94.4)	127 (91.4)	150 (96.1)	7.4 (0.4, 17.1)	**0.03**
	Line listing forms available in HF	60 (84.5)	78 (78.8)	81 (80.2)	95 (75.4)	114 (82.0)	122 (78.2)	6.3 (-5.4, 16.0)	0.27
	Case based forms available in HF	66 (93.0)	79 (79.8)	91 (90.1)	110 (87.3)	114 (82.0)	139 (89.1)	3.9 (-5.4, 11.0)	0.36
	Rumor logs available in HF	39 (54.9)	55 (55.6)	52 (51.5)	78 (61.9)	84 (60.4)	97 (62.1)	7.2 (-6.3, 20.8)	0.31
Data Analysis and Use	HF conducts data basic data analysis	28 (39.4)	49 (49.5)	41 (40.6)	51 (40.5)	63 (45.3)	99 (63.4)	24.0 (10.0, 36.7)	**0.0008**
	HF have current line graphs showing trends of priority diseases	5 (7.0)	11 (11.1)	16 (15.8)	29 (23.0)	43 (30.9)	72(46.1)	39.1 (27.8, 47.9)	**< 0.0001**
Outbreak notification and investigation	HF reported outbreak within 12 months	12 (16.9)	45 (45.5)	32 (31.7)	10 (7.9)	7 (5.0)	17(10.9)	6.0 (-4.2, 16.1)	0.31
	Outbreak notified to DHMT within 48 h*	11 (91.7)	29 (64.4)	29 (90.6)	9 (90.0)	5 (71.4)	15(88.2%)	3.5 (-24.7, 27.1)	0.09
Monitoring and Communication	Supervisory visits from DHMT (at least once every three months)	44 (62.0)	91 (91.9)	78 (77.2)	79 (62.7)	70 (50.4)	144 (92.3)	30.3(18.6, 42.4)	**< 0.0001**
	Mobile network available in HF	67 (94.4)	79 (79.8)	91 (90.1)	120 (95.2)	126 (90.6)	144 (92.3)	2.1 (-6.5, 8.4)	0.57

*Denominator is number of outbreaks reported in the preceding 12 months

HF: Health Facility; IDSR: Integrated Disease Surveillance and Response; HCW: Healthcare worker; DHMT: District Health Management Team

### Identification of priority IDSR diseases, conditions and events

**Fig. 2:**
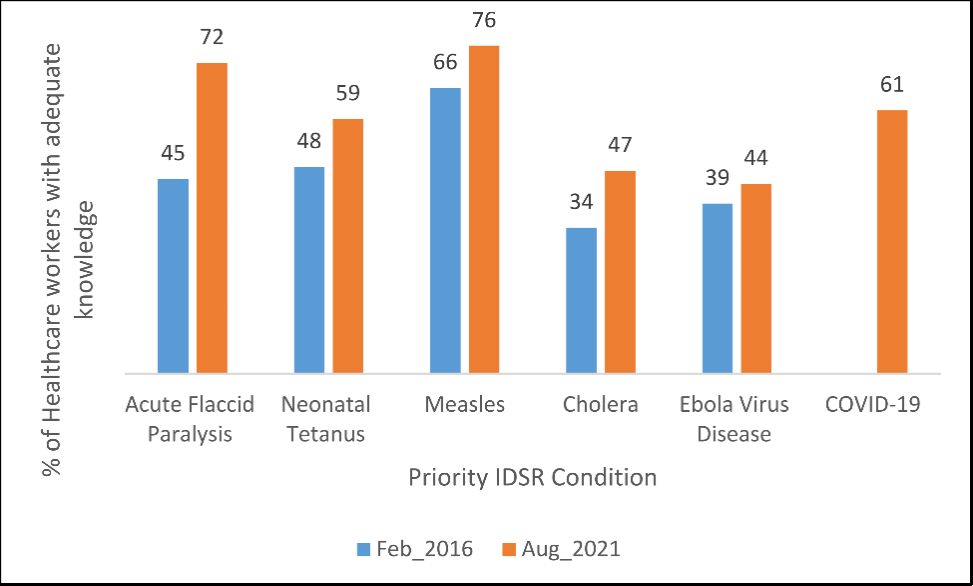
Knowledge of standard case definitions among interviewed healthcare workers, Sierra Leone, 2016 and 2021

The proportion of health facilities that displayed standard case definition posters in consultation rooms for use by health workers in identifying cases increased significantly from 76.1% to 2016 to 94.9% in 2021 (increase of 18.8% points; 95% CI 9.2, 30.2); P- value 0.002). The proportion of IDSR focal persons who conducted weekly active case search for priority diseases in the health facilities remained the same at 79% for both baseline and end line although it fluctuated across the years. There was positive trend in adequate knowledge of standard case definitions among health workers for five priority diseases that were assessed as shown in Fig. 2 (Acute flaccid paralysis, Neonatal tetanus, Measles, Cholera and Ebola Virus Disease). Additionally, COVID-19 was added in 2021 and 61% of the health workers had adequate knowledge on its case definition.

### Awareness and adherence to IDSR reporting requirements

Knowledge of IDSR reporting requirements was high and by the end line assessment in 2021, almost all interviewed health care workers correctly defined the epidemiologic week, zero reporting and reporting deadlines (Table 2). There was a significant improvement in the proportion of health facilities that submitted all the required surveillance reports (completeness of reporting) from 84.5% to 2016 to 96% in 2021(increase of 11.5% points; 95% CI 3.6, 21.9; P-value 0.003). During the same period, timeliness of IDSR reports improved from 80.3 to 92% (increase of 11.7% points; 95% CI 2.4, 22.9; P-value 0.01).

### Availability of IDSR reporting tools

Availability of the weekly IDSR reporting tool improved from 88.7% to 2016 to 96.1% in 2021 (increase of 7.4% points; 95% CI 0.4, 17.1; P-value 0.03). Availability of other reporting tools did not change significantly and were at 78%, 89% and 62% in 2021 for line listing forms, case-based reporting forms and rumour logbooks, respectively (Table 2).

### Data analysis and use at health facility level

There was significant improvement in data analysis and use in health facilities over the years. The proportion of health facilities that conducted basic data analysis improved from 39% at baseline to 63% at end line (increase of 24% points; 95% CI 10.0, 36.7; P-value 0.0008). The proportion of health facilities with current line graphs showing trends in occurrence of priority diseases increased significantly from 7 to 46.1% (increase of 39.1% points; 95% CI 27.8, 47.9; P-value < 0.0001) (Table 2).

### Outbreak detection and notification

The proportion of health facilities that had identified an outbreak within 12 months of the assessment did not change significantly and was 16.9% at baseline compared to 10.9% at end line (P-value 0.31). This was mostly because there were no outbreaks to detect and not for lack of detection capacity. The proportion of identified outbreaks that were notified to the District Health Management Teams within 48 h did not change significantly and were 91.7% at baseline compared to 88.2% at end line (P-value 0.09) (Table 2).

### Feedback, monitoring and communication

Quarterly supervisory visits by DHMTs to the health facilities improved from 62% at baseline to 92% (P-value < 000.1) at end line although it fluctuated for other visits (Table 2). Mobile network connectivity remained high throughout the period and 92% of the health facilities assessed at end line had connectivity.

### Availability of routine immunization services and commodities for management of Malaria

The proportion of health facilities providing routine immunization services was above 90% in all the assessments with the end line being 96%. The number of immunizing health facilities with functional refrigerators significantly improved from 49.2% at baseline to 66.7% (P-value 0.02) although it fluctuated for other visits (Table 3). Immunizing health facilities with updated temperature monitoring charts significantly improved from 34 to 81% (P-value < 0.0001) while health facilities with all required basic antigens (vaccines) available at the time of the assessment improved from 48 to 83% (P-value < 0.0001). At the end line assessment, there were only 6.7% health facilities with Vaccine Vial Monitor (VVM) at stage three or four which is considered as excessive exposure to high temperatures (Table 3). Malaria management commodities including first line anti-malarial drugs, rapid diagnostic test kits and insecticide treated nets were available in most of the health facilities throughout the assessment period although there was a significant drop for first line anti-malarial drugs and rapid diagnostic test kits at end line assessment in August 2021, mostly due to disruptions associated with COVID-19 (Table 3).

**Table 3:** Assessing routine immunization, malaria commodities and diagnostic capacity in health facilities, Sierra Leone, 2016–2021

Programmatic Area	Variable	Feb_2016	July_2016	May_2017	Aug_2018	Feb_2019	Aug_2021	End Line vs. Baseline	P-value
		(N = 71) n (%)	(N = 99) n (%)	(N = 101) n (%)	(N = 126) n (%)	(N = 139) n (%)	(N = 156) n (%)	Difference (95 CI)	
**Vaccination**	Health facilities providing routine Immunization Services	65 (91.5)	95 (96.0)	96 (95.0)	120 (95.2)	135 (97.1)	150 (96.2)	4.7 (-1.5, 13.7)	0.14
	Health facilities with functional refrigerator†	32 (49.2)	70 (73.7)	72 (75.0)	71 (59.2)	88 (65.2)	100 (66.7)	17.5 (3.3, 31.2)	**0.0157**
	Health facilities with updated temperature monitoring chart††	11 (34.4)	16 (22.9)	32 (44.4)	29 (40.8)	37 (42.0)	81 (81)	46.6 (27.2,62.0)	**< 0.0001**
	Health facilities with all required basic antigens available at the time of the assessment†	31(47.7)	55(57.9)	60(62.5)	86 (71.7)	103 (76.3)	125 (83.3)	35.6 (21.9, 48.3)	**< 0.0001**
	Heath facilities with expired antigens†	2 (3.1)	3 (3.2)	1 (1)	1 (0.8)	2 (1.5)	2 (1.3)	1.8 (-2.3, 9.3)	0.37
	Health facilities with Vaccine Vial Monitor at stage three and four†	14 (21.5)	16 (16.8)	14 (14.6)	8 (6.7)	9 (6.7)	10 (6.7)	14.8 (5.1, 26.6)	**0.002**
**Malaria Commodities**	Health facilities with 1st line anti-malaria drugs	69 (97.2)	96 (97.0)	99 (98.0)	120 (95.2)	135 (97.1)	120 (76.9)	20.3 (11.2, 27.7)	**0.0002**
	Health Facilities with rapid diagnostic kits for diagnosis of malaria	67 (94.4)	89 (89.9)	98 (97.0)	120 (95.2)	134 (96.4)	119 (76.3)	18.1 (8.1, 26.1)	**0.001**
	Health facilities with insecticide treated nets	55 (77.5)	92 (92.9)	97 (96.0)	111 (88.1)	132 (95.0)	139 (89.1)	11.6 (1.6, 23.3)	**0.0218**

†Denominator is number of health facilities providing immunization services

†† Denominator is number of health facilities with a functional fridge

## Discussion

Through the support supervision data collected and analysed after each visit to the districts, the country was able to monitor trends of key IDSR indicators. In general, health facilities performed well over the years in several assessment areas (Tables 2 and 3). However, there were also some other areas that did not improve much due to several challenges including inadequate funding and COVID-19 pandemic. The funding challenge was partly resolved by sharing the supervision feedback with stakeholders who could provide financial support to address some of the challenges. For example, WHO and other development partners supported the MoHS to print and distribute surveillance tools to health facilities and to conduct refresher trainings for health workers focusing on case definitions of priority diseases, conditions and events listed in the IDSR technical guidelines. However, as the findings of the availability of surveillance tools shows (Table 2), only the weekly reporting tool had become more available over the years compared to the other tools (line listing forms, case-based reporting forms and rumour logbooks). This is because the weekly tool is the one more frequently used compared to the others and hence development partners were more likely to support in its printing.

Surveillance data is more accurate in measuring burden of disease if it is representative and timely [9]. IDSR report completeness (proportion of health facilities submitting reports) and timeliness improved during the review period and may be partly attributed to availability of IDSR focal persons in most health facilities who were familiar with IDSR reporting obligations and were equipped with the tools necessary for reporting. Lack of IDSR focal persons and unavailability of technical guidelines are often associated with low weekly IDSR reporting rates and timeliness [17]. Gradual migration from paper based to electronic reporting of IDSR data in Sierra Leone at the health facility level starting mid-2017 may have improved timeliness of IDSR reports by reducing report transmission time [18]. Reporting completeness and timeliness rates in these assessments are comparable to those found in a similar assessment in Uganda [19].

Over the years, there was overall improvement in knowledge of case definitions for priority conditions among health workers which could have been due to increased availability of case definition posters in health facilities. However, the proportion of health workers with adequate knowledge of the case definitions was less than 80% in all the diseases sampled which means that not all health workers were using the case definition posters (Fig. 2). For Ebola Virus Disease, the suboptimal knowledge of the case definition may have been due to changes in case definitions (from outbreak case definition to a routine surveillance case definition). Low knowledge on the case definition for Cholera was likely because the country had not recorded any case since the last outbreak in 2013.

Syndromic surveillance is important in low resource settings where laboratory confirmation is not always readily available as is currently the case in Sierra Leone. Inadequate knowledge on case definitions leads to low levels of suspicion or misclassification of cases. Hence, it is important that healthcare workers use clinical signs and symptoms as depicted in the standard case definition posters to suspect cases and initiate investigation. The Ministry of Health and Sanitation must therefore do more to engage the health workers in all health facilities to ensure that they read and master the case definitions for the various diseases since the posters are available in most health facilities.

Data analysis at the health facilities improved markedly over time from 2016 to 2021 (Table 2) and the reasons given by the health workers for this improvement were mainly training on data analysis skills, provision of data analysis graphs and tablets for reporting. In deed these are important elements in data analysis as lack of computers and technical capacity has been reported in other countries as being responsible for poor data analysis [19]. Limited laboratory diagnostic capacity was observed even for organisms such as Vibrio *cholerae* that have caused large magnitude epidemics in Sierra Leone in the past [20]. This limits the contribution of laboratories to outbreak detection, guiding case management and monitoring trends of priority diseases as specimen referral increases turnaround time for results.

While routine vaccination services were available in most health facilities, gaps were observed in maintenance of cold chain that could have compromised the quality of vaccines. It is worth noting that the gaps reduced in subsequent assessments with significant improvement in the number of health facilities with all basic vaccines and functional cold chain equipment. Availability of first line antimalarial drugs and rapid diagnostic kits for malaria detection dropped during the end line assessment in 2021 and this could have been attributed to the COVID-19 pandemic which has caused constraint on available resources including for malaria [21].

This study had a few limitations. Even though two national IDSR support supervision visits were planned each year, only one was conducted annually except in 2016 when two were made. This was mostly due to competing priorities or resource constraints. While two visits per year would have provided us with better trends, it did not affect the quality of the data. Support supervision was however conducted by the districts on a quarterly basis although the comprehensive electronic checklist was not always used and this data could therefore not be included. Additionally, due to the COVID-19 pandemic, no national IDSR support supervision was conducted in 2020 due to travel restrictions to the districts for most part of the year. However, there were other visits made to the districts as part of the response to the pandemic.

## Conclusion

The IDSR system is now well established in Sierra Leone. The support supervision visits that were done using an integrated electronic tool contributed to health systems strengthening through longitudinal tracking of core IDSR indicators and other program indicators such as essential malaria commodities and availability and status of routine vaccines. The feedback that was provided to all levels of healthcare including the national program and development partners supported to address the gaps identified and hence improved performance over the years. MoHS should entrench the supportive supervision approach by the national and district teams using electronic tools to assure sustained monitoring of IDSR and other programs.

## Data Availability

The datasets analyzed during this assessment are available from the corresponding author upon reasonable request.

## List of abbreviations

AfDB: African Development Bank.
CHC: Community Health Centre.
CHP: Community Health Post.
DFID: Department for International Development.
DHMT: District Health Management Team.
DMO: District Medical Officer.
e-IDSR: Electronic Integrated Disease Surveillance and Response system.
IDSR: Integrated Disease Surveillance and Response.
IHR: International Health Regulations.
MCHP: Maternal Child Health Post.
MoHS: Ministry of Health and Sanitation.
MPTF: Multi partners Trust Fund.
ODK: Open Data Kit.
US-CDC: United States Centers for Disease Control and Prevention.
VVM: Vaccine Vial Monitor.
WCO: WHO Country Office.
WHO: World Health Organization.
